# Ewing sarcoma of the first metacarpal: a rare case report with thumb-sparing resection and fibular graft reconstruction

**DOI:** 10.3389/fsurg.2026.1833919

**Published:** 2026-04-20

**Authors:** Aouinti Mohamed Nizar, Sahar Ben Ammar, Walid Saied, Hajer Ben Mansour, Ahmed Hamdi, Henda Rais, Sami Bouchoucha, Rim Boussetta

**Affiliations:** 1 Pediatric Orthopeadic Departement, Hospital Children Béchir Hamza, Tunis, Tunisia; 2 Institut Salah Azaiez, Tunis, Tunisia; 3 Center for Traumatology and Major Burns, Ben Arous, Tunis, Tunisia

**Keywords:** dominant thumb, Ewing sarcoma, fibular graft, first metacarpal, limb-sparing surgery, pediatric bone tumor

## Abstract

**Background:**

Ewing sarcoma is a malignant primary bone tumor that predominantly affects the long bones and pelvis of children and adolescents. Involvement of the hand is exceptionally rare, particularly at the level of the first metacarpal. When the dominant thumb is affected, treatment becomes especially challenging due to the critical functional role of this structure.

**Case presentation:**

We report the case of an 11-year-old right-handed boy who presented with a painful swelling of the right thumb. Imaging revealed an aggressive osteolytic lesion of the first metacarpal with soft tissue extension. Histology confirmed Ewing sarcoma. After neoadjuvant chemotherapy according to the EuroEWing 2012 protocol, Thumb-sparing resection was performed, including the trapeziometacarpal and metacarpophalangeal joints. Reconstruction was achieved using a non-vascularized fibular autograft. Despite a poor histological response, surgical margins were tumor-free. Adjuvant chemotherapy and radiotherapy were administered. At one-year follow-up, there was no local recurrence, with satisfactory functional outcome of the dominant hand.

**Conclusion:**

Ewing sarcoma of the first metacarpal is exceedingly rare. Limb-sparing surgery with fibular graft reconstruction may represent a valid alternative to amputation in carefully selected pediatric patients, even in cases of limited histological response, provided that oncological principles are respected.

## Introduction

Ewing sarcoma is the second most common primary malignant bone tumor in children and adolescents, typically arising in the diaphysis of long bones, the pelvis, and the chest wall (1). Involvement of the hand is exceptionally rare, accounting for less than 1% of cases, with metacarpal localization being particularly uncommon (2, 3). Among these, the first metacarpal represents an exceedingly rare site, with only isolated cases reported in the literature (4).

The thumb plays a central role in hand biomechanics, contributing to nearly 40% of overall hand function through opposition, pinch, and precision grip. This role becomes even more critical when the dominant hand is involved, especially in children, where impairment may significantly affect development, autonomy, and long-term quality of life. Consequently, malignant tumors of the dominant thumb present a major therapeutic dilemma, requiring a balance between oncological safety and functional preservation.

Management of Ewing sarcoma relies on a multimodal approach combining systemic chemotherapy, surgical resection, and, in selected cases, radiotherapy (5). Historically, hand involvement often led to radical treatment such as amputation to ensure local control (6). However, advances in chemotherapy protocols and reconstructive techniques have progressively enabled limb- and function-sparing strategies in selected patients without compromising oncological outcomes (7).

We report a rare case of Ewing sarcoma of the first metacarpal of the right dominant thumb in a child, treated by wide tumor resection and reconstruction using a fibular autograft. To our knowledge, this is the only reported pediatric case of first metacarpal Ewing sarcoma managed with this reconstructive strategy.

## Case report

### Clinical presentation

The patient was an 11-year-old right-handed boy, with no prior medical or surgical history. He had no history of trauma, infection, or previous treatment involving the affected hand. There was no family history of malignancy.

From a psychosocial perspective, the patient was fully independent in daily activities and actively engaged in school and recreational activities. Preservation of the dominant thumb was therefore considered essential for maintaining autonomy and quality of life.

He presented with a progressively painful swelling of the right thumb extending to the thenar region. Symptoms had evolved over several weeks without trauma or signs of infection. Physical examination revealed a firm, tender, poorly defined mass fixed to the underlying bone, causing limitation of active and passive thumb motion due to pain. There were no skin changes, inflammatory signs, or neurovascular deficits.

### Imaging findings

**Figure 1:**
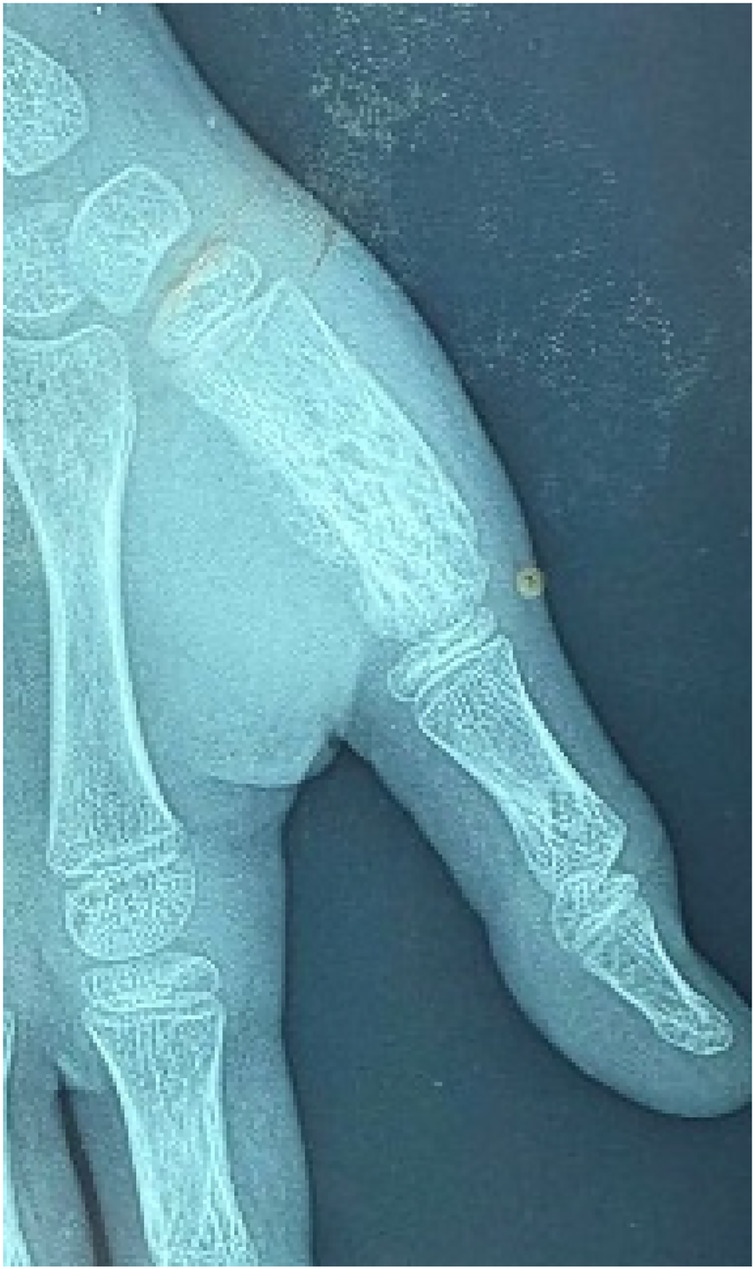
Preoperative radiograph showing osteolytic lesion of the first metacarpal.

Plain radiographs demonstrated an ill-defined osteolytic lesion of the first metacarpal, associated with cortical thinning, irregular margins, and adjacent soft tissue swelling (Figure 1).

**Figure 2:**
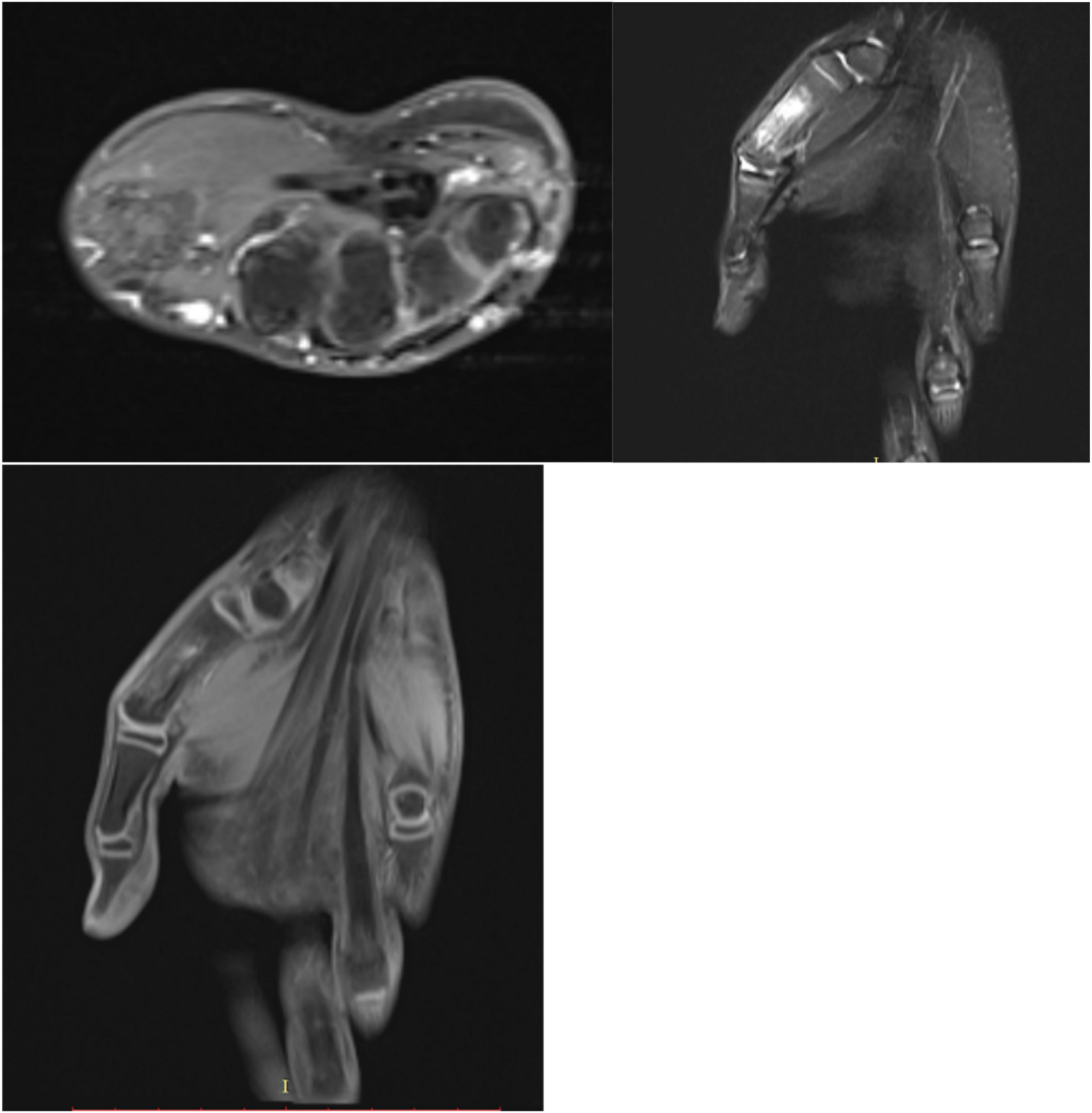
Preoperative MRI demonstrating diaphyseal tumor with thenar extension.

Magnetic resonance imaging (MRI) revealed a diaphyseal tumor of the first metacarpal measuring 24 × 24 × 30 mm, with extension into the thenar musculature. The lesion showed low-to-intermediate signal intensity on T1-weighted images and heterogeneous high signal on T2-weighted images (Figure 2).

Bone scintigraphy demonstrated isolated hyperfixation of the first metacarpal with no other skeletal involvement. Initial Thoraco-abdominopelvic computed tomography (CT) revealed three small pulmonary nodules (3 mm each), raising suspicion for metastatic disease.

The differential diagnosis of an aggressive osteolytic lesion of the metacarpal in a pediatric patient includes osteomyelitis, Langerhans cell histiocytosis, osteosarcoma, and other small round cell tumors such as lymphoma.

### Histological diagnosis

A percutaneous biopsy confirmed the diagnosis of Ewing sarcoma, showing sheets of small round blue cells consistent with this entity, supported by immunohistochemical analysis.

Imaging findings combined with histopathological and immunohistochemical analysis were essential to establish the definitive diagnosis of Ewing sarcoma.

### Oncological treatment

Pulmonary nodules were not biopsied due to their small size and were managed within the framework of systemic chemotherapy.

The patient was treated according to the EuroEWing 2012 protocol, receiving alternating cycles of VDC (vincristine, doxorubicin, cyclophosphamide) and IE (ifosfamide, etoposide).

- VDC regimen: vincristine 1.5 mg/m^2^ (maximum 2 mg) on day 1, doxorubicin 37.5 mg/m^2^/day on days 1–2 (The cumulative dose of doxorubicin should not exceed 600 mg/m^2^), cyclophosphamide 1,200 mg/m^2^ on day 1.
- IE regimen: ifosfamide 1,800 mg/m^2^/day and etoposide 100 mg/m^2^/day for five consecutive days with Mesna: 600 mg/m^2^ to prevent hemorrhagic cystitis.

A total of five cycles of VDC and four cycles of IE were administered at 15-day intervals with G-CSF support at the dose of to prevent neutropenia GIII and IV. Treatment was generally well tolerated, with occasional delays due to grade 2 neutropenia.

Interim MRI demonstrated significant tumor shrinkage and marked reduction of soft tissue involvement.

Adjuvant treatment combining chemotherapy and radiotherapy was reintroduced two months following surgery:

VC cycles: vincristine 1.5 mg/m^2^ (max 2 mg) on day 1 and cyclophosphamide 1,200 mg/m^2^ on day 1, 2 cycles.

IE cycles: ifosfamide 1,800 mg/m^2^/day and etoposide 100 mg/m^2^/day for five consecutive days, 3 cycles.

Doxorubicin was not included in the treatment plan to avoid the risk of radiation effects and serious skin toxicity.

Radiotherapy was administered concurrently with chemotherapy, delivering a total dose of 45 Gy in 25 fractions, at a schedule of five fractions per week.

### Surgical management and reconstruction

After approximately five months of systemic therapy, wide *en bloc* resection was performed. The resection included the entire first metacarpal, with a proximal intra-articular resection at the trapeziometacarpal joint and a distal extra-articular resection at the base of the proximal phalanx, ensuring oncologically safe margins. The procedure was carried out through transmuscular planes in accordance with oncological surgical principles.

Particular attention was paid to tendon management. The extensor pollicis longus and abductor pollicis longus tendons were preserved, while the flexor tendons were resected *en bloc* with the tumor due to their proximity and potential involvement.

Reconstruction was achieved using a non-vascularized fibular autograft measuring approximately 5 cm in length, allowing restoration of thumb length and alignment. Fixation was performed using two mini-fragment plates: a distal straight plate and a proximal T-shaped plate, both secured with cortical screws, providing stable fixation.

**Figure 3:**
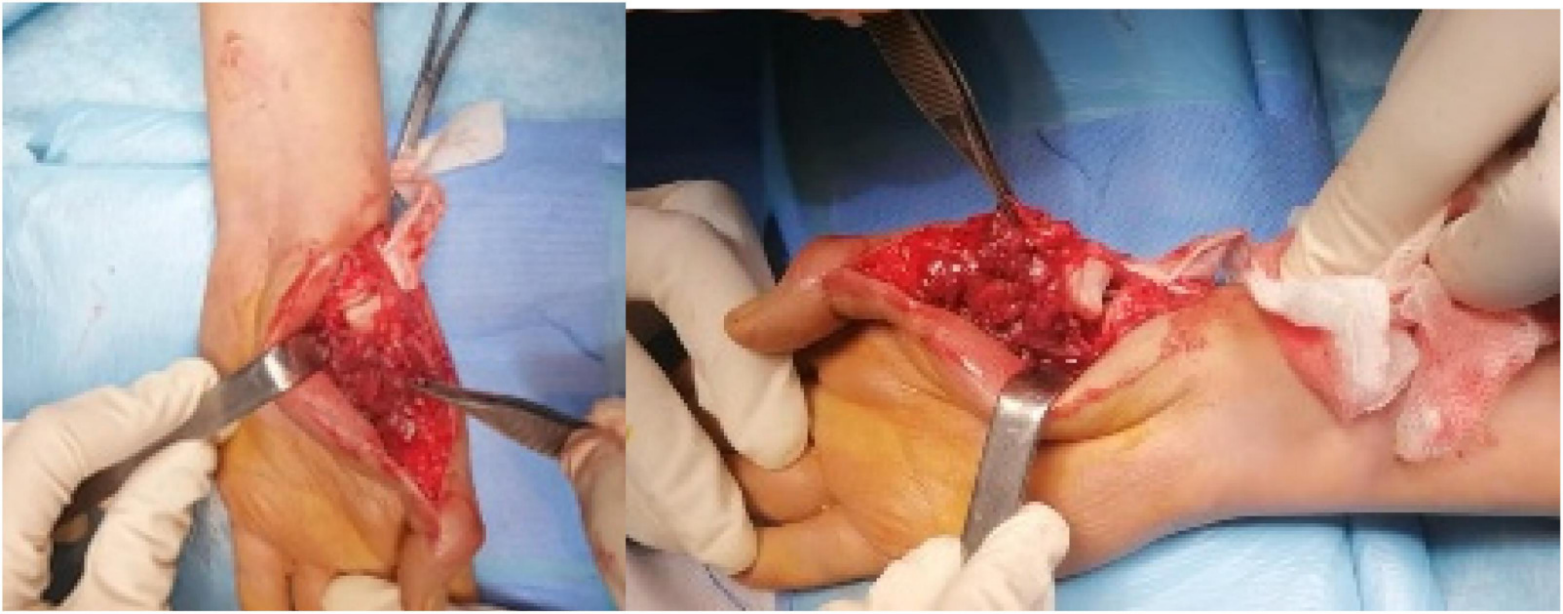
Intraoperative view and resection specimen.

The abductor pollicis longus tendon was reinserted at the graft junction. Adequate soft tissue coverage was obtained using the residual thenar musculature (Figure 3).

Histological examination confirmed tumor-free (R0) margins,

### Pathological findings

**Figure 4:**
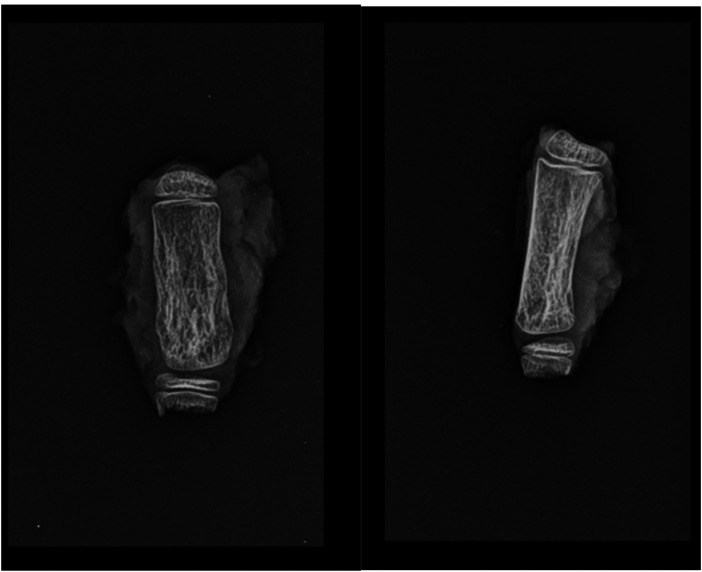
Surgical specimen.

Histological examination of the resection specimen revealed approximately 90% viable tumor tissue, indicating a poor histological response to chemotherapy. There was no cortical breakthrough, no infiltration of surrounding soft tissues, and surgical margins were free of tumor. According to the Ouvos grading system, the tumor was classified as grade A (Figure 4).

### Postoperative course and follow-up

Postoperative immobilization with a forearm-thumb cast was maintained for 45 days. The postoperative course was uneventful.

A postoperative thoraco-abdominopelvic CT scan demonstrated complete resolution of these nodules, suggesting either benign etiology or complete response to chemotherapy.

Two months after surgery, adjuvant chemotherapy was resumed, consisting of two cycles of VC and three cycles of IE, combined with radiotherapy. Chemotherapy doses were reduced by 20% due to recurrent grade 4 hematological toxicity.

**Figure 5:**
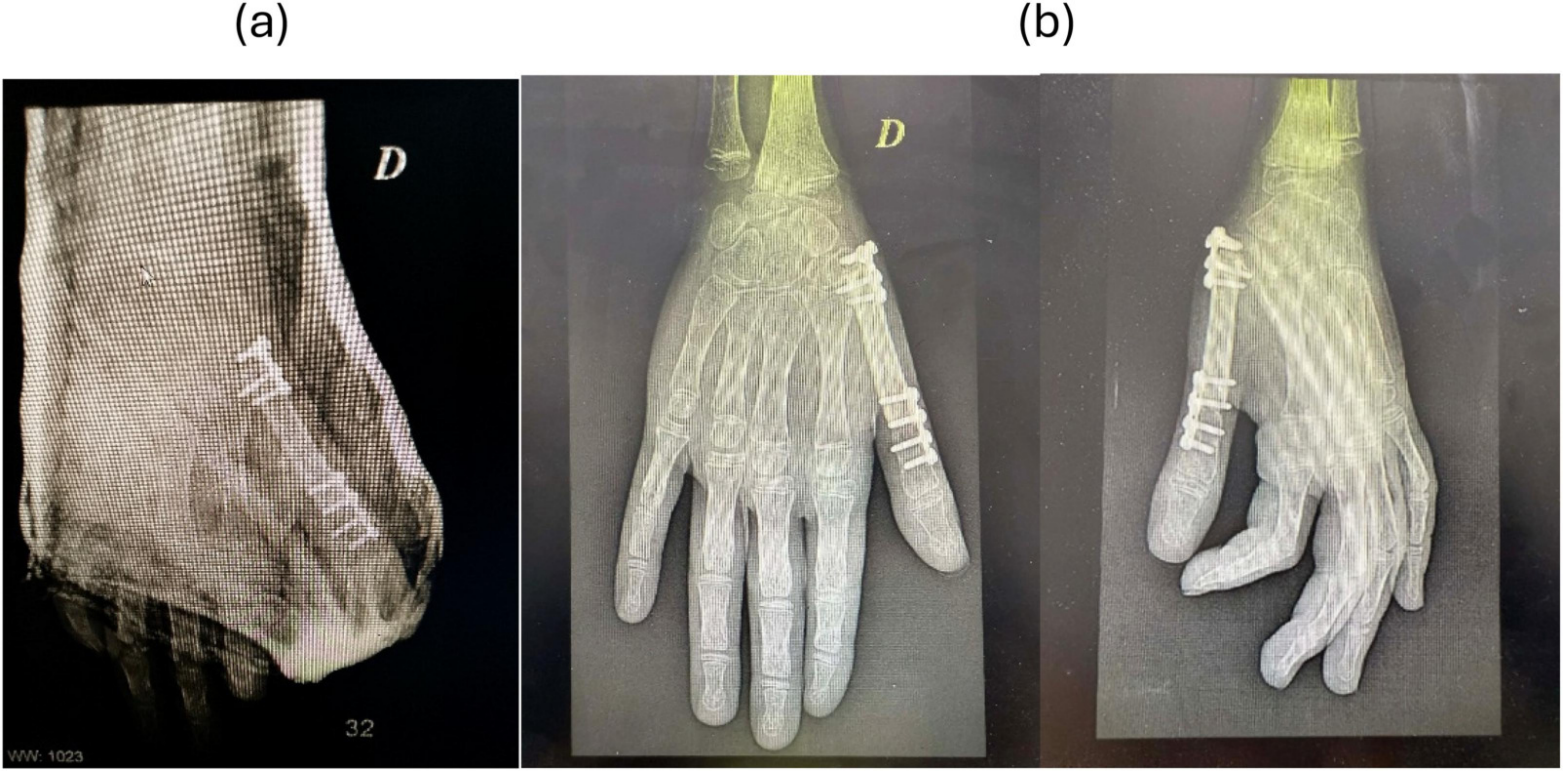
Postoperative radiograph **(a)** and at one-year follow-up **(b)**.

At one-year follow-up, the patient showed no evidence of local recurrence or disease progression. Radiographs demonstrated satisfactory graft integration (Figure 5). Functional evaluation of the thumb demonstrated moderate abduction, while active flexion was absent, consistent with the intraoperative resection of the flexor tendons.

Despite this limitation, preservation of the extensor and abductor mechanisms allowed maintenance of a functional lateral pinch and satisfactory thumb positioning.

Global hand function was preserved, with the patient able to perform basic grasping tasks independently.Functional outcome was supported by an estimated DASH score of approximately 25/100, indicating mild to moderate functional impairment.

**Figure 6:**
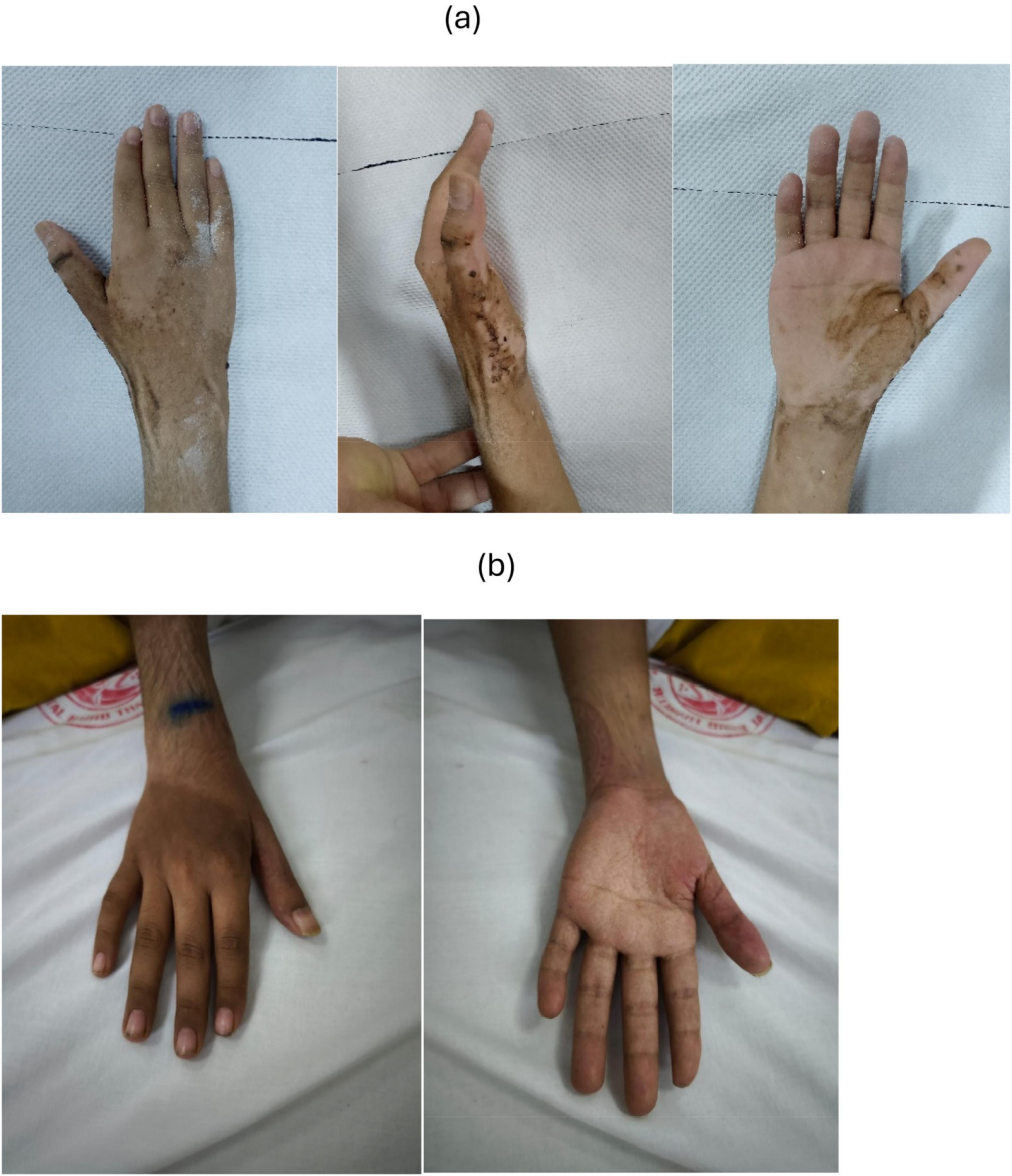
Clinical appearance of the hand after removing cast **(a)** at one-year follow-up **(b)**.

The patient and his family expressed satisfaction with the preservation of thumb function (Figure 6), which allowed independence in daily activities.

### CARE-compliant timeline of clinical course

- Initial presentation: Progressive painful swelling of the right thumb
- Imaging: x-ray, MRI confirming aggressive lesion of first metacarpal
- Staging: Thoraco-abdominopelvic CT showing three small pulmonary nodules
- Biopsy: Histological confirmation of Ewing sarcoma
- Neoadjuvant chemotherapy: EuroEWing 2012 protocol (VDC/IE)
- Reassessment: Tumor regression on MRI
- Surgery: Wide resection and fibular graft reconstruction
- Postoperative staging: CT scan showing complete resolution of pulmonary nodules
- Adjuvant therapy: Chemotherapy ± radiotherapy
- Follow-up: 1 year, no local recurrence or metastasis, functional preservation

## Discussion

Ewing sarcoma of the hand is exceptionally rare, with metacarpal involvement representing a very small subset of reported cases (2, 3). Localization to the first metacarpal is even more uncommon and presents unique therapeutic challenges due to the essential biomechanical role of the thumb (4). When the dominant hand is affected, these challenges are further amplified, particularly in pediatric patients.

Historically, amputation was considered the safest oncological option for Ewing sarcoma of the hand (6). However, improvements in chemotherapy protocols and surgical techniques have enabled limb-sparing strategies in selected cases without compromising local control (5, 7). In the present case, the involvement of the dominant thumb strongly influenced the decision toward a conservative surgical approach.

Despite a favorable radiological response to neoadjuvant chemotherapy, histological examination revealed a poor response, with approximately 90% viable tumor tissue. Poor histological response is a recognized adverse prognostic factor in Ewing sarcoma and usually warrants treatment intensification (6). In our case, the absence of soft tissue infiltration, lack of cortical breach, and achievement of wide tumor-free margins allowed safe reconstruction, supported by aggressive adjuvant therapy. The decision to preserve the thumb despite poor histological response remains controversial. While negative margins were achieved, this approach may carry a higher risk of recurrence compared to amputation, necessitating strict follow-up.

Reconstruction after metacarpal resection remains challenging. Reported techniques include bone cement spacers, allografts, toe transfers, and vascularized or non-vascularized fibular grafts (8–10). Fibular autograft reconstruction offers biological integration, structural strength, and adaptability, making it particularly suitable for pediatric patients (11). In this case, it allowed restoration of thumb length and stability while preserving surrounding musculotendinous structures.

To the best of our knowledge, very few cases have been reported. This case illustrates that, even in the context of limited histological response, conservative surgery may be justified when oncological principles are respected and functional preservation is paramount.

## Conclusion

Ewing sarcoma of the first metacarpal in children is exceedingly rare, particularly when involving the dominant thumb. This case demonstrates that Thumb-sparing resection combined with fibular autograft reconstruction can achieve satisfactory oncological control and functional outcome. Multidisciplinary management and individualized treatment planning are essential in such rare and complex localizations.

## Data Availability

The raw data supporting the conclusions of this article will be made available by the authors, without undue reservation.
